# Acyl-CoA-binding protein (*ACBP*) genes involvement in response to abiotic stress and exogenous hormone application in barley (*Hordeum vulgare* L.)

**DOI:** 10.1186/s12870-024-04944-6

**Published:** 2024-04-02

**Authors:** Huayu Chang, Minhu Ma, Mingzhou Gu, Shanshan Li, Mengrun Li, Ganggang Guo, Guofang Xing

**Affiliations:** 1 Hou Ji Laboratory in Shanxi Province, College of Agriculture, Shanxi Agricultural University, Taiyuan, Shanxi 030031 China; 2 Key laboratory of Grain Crop Genetic Resources Evaluation and Utilization (MARA), State Key Laboratory of Crop Gene Resources and Breeding, Institute of Crop Sciences, Chinese Academy of Agricultural Sciences (ICS-CAAS), Beijing, 100081 China

**Keywords:** Barley stress response, Acyl-CoA-binding protein (ACBP), Gene expression pattern

## Abstract

**Background:**

Acyl-CoA-Binding proteins (ACBPs) function as coenzyme A transporters and play important roles in regulating plant growth and development in response to abiotic stress and phytohormones, as well as in membrane repair. To date, the *ACBP* family has not been a comprehensively characterized in barley (*Hordeum vulgare* L.).

**Results:**

Eight ACBP genes were identified in the barley genome and named as *HvACBP1–8*. The analysis of the proteins structure and promoter elements of *HvACBP* suggested its potential functions in plant growth, development, and stress response. These *HvACBPs* are expressed in specific tissues and organs following induction by abiotic stressors such as drought, salinity, UV-B exposure, temperature extremes, and exposure to exogenous phytohormones. The HvACBP7 and HvACBP8 amino acid sequences were conserved during the domestication of Tibetan Qingke barley.

**Conclusions:**

Acyl-CoA-binding proteins may play important roles in barley growth and environmental adaptation. This study provides foundation for further analyses of the biological functions of HvACBPs in the barley stress response.

**Supplementary Information:**

The online version contains supplementary material available at 10.1186/s12870-024-04944-6.

## Background

Lipids are essential components of cell membranes and play pivotal roles in the cellular framework [1–3]. They provide energy for intracellular activity and sites for physiological processes such as photosynthesis, signal recognition, and transmission. They also act as signaling molecules in growth, development, and stress responses [4–6]. Key proteins involved in non-vesicular lipid transportation include lipid transfer proteins, ATP-binding cassette transporters, and acyl-coenzyme A-binding proteins (ACBPs) [7]. Compared with lipid transfer proteins and ATP-binding cassette transporters, ACBPs exhibit a wider range of lipid capabilities, allowing for broader subcellular localization. In addition, they can interact with long acyl-coenzyme A (acyl-CoA) moieties [8].

ACBPs are classified into four subfamilies (Class I–IV) based on their functional domains and relative molecular masses. Class I (small ACBPs) consists of low-molecular-weight ACBPs with only one ACBP domain. Class II (ANK-ACBPs) consists of higher-molecular-weight ACBPs and N-terminal ACBP domains coupled with C-terminal ankyrin domains [9, 10]. Class III (large ACBPs) includes various higher-molecular-weight ACBPs with one ACBP domain [11]. Class IV (Kelch-ACBPs) comprises higher-molecular-weight ACBPs with N-terminal ACBP domains and multiple C-terminal Kelch structural domains [12, 13].

As with other lipid transport proteins, the functions of ACBPs are influenced by their subcellular localization. All Class I ACBPs, including AtACBP6 [14], OsACBP1–3 [15], ChACBP1 [16], and HbACBP1 [9], are localized in the cytoplasm. Class II ACBPs, including AtACBP1/2 [17] and OsACBP4 [15], are localized in the cell membrane and endoplasmic reticulum, whereas HbACBP2 is localized in the endoplasmic reticulum [9]. The subcellular localization of Class III ACBPs is diverse: AtACBP2 is found in the peroxisomes, cell membranes, and endoplasmic reticulum around the Golgi apparatus complex [18]; OsACBP5 is localized in the peroxisomes [15]; and VfACBP3A and VfACBP3B are localized in the endoplasmic reticulum [19]. Among Class IV ACBPs, AtACBP4 and AtACBP5 are localized in the cytoplasm [20], whereas OsACBP6 is localized in the peroxisome [15].

In *Arabidopsis*, *AtACBP* expression varies widely among the different organs (roots, cotyledons, flowers, fruits, seedlings, and mature plants) [13]. *AtACBP1–2* expression is highest in the siliques and mature seeds [21–23], whereas *AtACBP3* is highly expressed in the pistils [11, 24, 25]. *AtACBP5* and *AtACBP6* are highly expressed in pollen, microspores, and trichome cells [2, 3, 14, 20, 26]. In rice, *ACBPs* are expressed in the roots, stems, leaves, and seeds, with the highest expression in the leaves and the lowest expression in the roots and stems [27, 28].

ACBPs have been studied for their roles in plant growth, development, and stress responses in model plants such as *Arabidopsis* and rice. In *Arabidopsis*, overexpression of *AtACBP6* enhances cold tolerance by upregulating the expression of phospholipase Dδ and increasing the levels of phosphatidic acid. AtACBP6 has binding affinity for various types of phosphatidylcholines, suggesting its role in phosphatidylcholine transport [14, 29]. *AtACBP6* knockout affects the seed oil content and lipid composition, leading to a reduced seed weight [30]. Similarly, in rice, overexpression of *OsACBP2* results in an increased grain size and oil content [31]. The expression of *OsACBP1–3* is suppressed under cold stress [27]. In *Arabidopsis*, *AtACBP1* and *AtACBP2* respond to cold, drought, hypoxia, and heavy metal stresses by maintaining a pool of membrane-associated acyl groups that are essential for early embryonic development [17, 20, 32, 33]. *AtACBP3* regulates autophagy-mediated leaf senescence and hypoxia responses, and its overexpression upregulates pathogenesis-related proteins and the salicylic acid pathway, thus resulting in improved pathogen resistance [25, 34]. Mutations in *AtACBP4–6* alter acyl-CoA expression, affecting both seed germination and pollen tube growth [2, 30]. *AtACBP4* responds to heavy metals, ethylene, methyl jasmonate (MeJA), and pathogen infections [35]. In rice, *OsACBP4* is responsive to salt and drought stress, and its overexpression significantly improves salt tolerance [27, 31]. *OsACBP5* overexpression enhances resistance to various pathogen types via the jasmonic acid and salicylic acid pathways [36, 37]. *OsACBP6* responds to physical damage, influences growth, participates in indole-3-butyric acid oxidation, and contributes to jasmonic acid biosynthesis [15, 27, 38]. In soybeans, *GmACBP3* and *GmACBP4* overexpression enhances lipoxygenase activity and salt tolerance [39]. These studies emphasize the critical role of ACBPs in plant biology, lipid metabolism, signaling pathways, and adaptive stress responses. However, there is a lack of research on the ACBP family of barley.

We comprehensively characterized the ACBP family in barley (*Hordeum vulgare* L.) and identified *HvACBPs* in barley using genome-wide bioinformatics analysis. Predicted the promoter elements to determine the changes in the expression of *HvACBPs* in response to abiotic stresses (temperature extremes, elevated salinity, drought, and UV exposure) and phytohormones indole-3-acetic acid (IAA), abscisic acid (ABA), and MeJA. This study provides a basis for the further in-depth exploration of the functional roles of *HvACBPs* to understand their complex functions in plant growth, development, and responses to environmental stimuli.

## Results

### Identification of barley ACBP gene family and phylogenetic and collinearity analysis

An extensive search for prior studies of ACBP in rice and *Arabidopsis* was conducted using public and private barley genomic and transcriptomic databases. Eight HvACBP genes were identified in the Morex reference genome. ACBP genes are distributed on all barley chromosomes, except for 3 H and 6 H. Additionally, *HvACBP3* and *HvACBP4* are tandem repeat genes on 2 H.

**Fig. 1:**
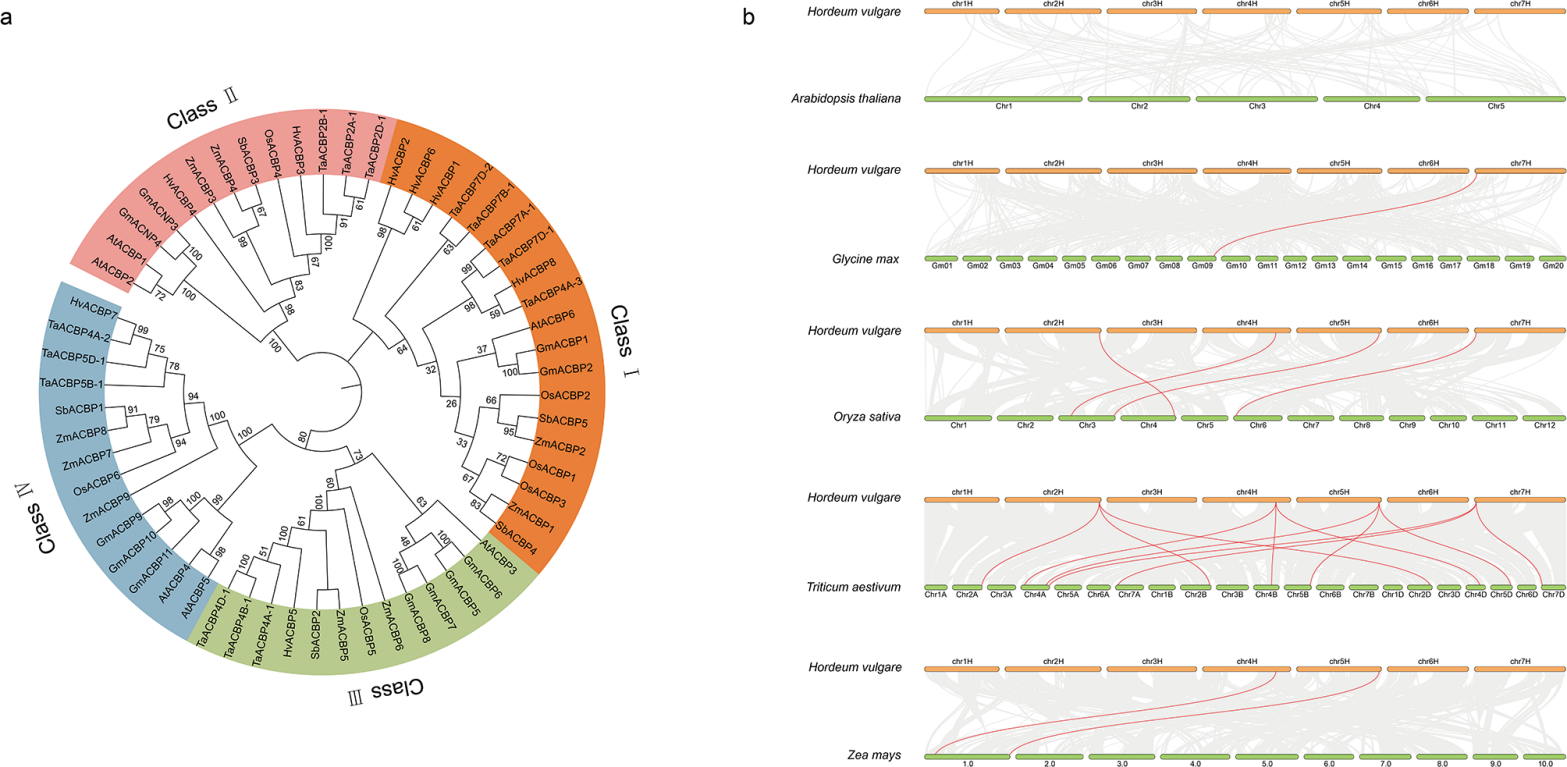
Phylogenetic analysis and collinearity analyses of ACBP in *Arabidopsis thaliana*, *Glycine max*, *Oryza sativa*, *Triticum aestivum*, *Zea mays*, *Sorghum bicolor*, and *Hordeum vulgare* (**a**) Phylogenetic tree of ACBP genes. The full-length sequence of the ACBP proteins was used for sequence alignment and phylogenetic analysis. Blocks of different colors indicate distinct subgroups, whereas in same color are indicated within a subgroup. (**b**) Collinear *ACBP* blocks in barley and related species. Gray lines in the background indicate collinear blocks within *Hordeum vulgare* and other plant genomes; collinear *ACBP* gene pairs are colored in red

To analyze the evolutionary relationships of ACBP genes among different species, 59 ACBP protein sequences from seven crops were used to construct a phylogenetic tree. The genes were classified into the four ACBP Classes (Fig. 1a). Based on comparison with genes in rice and *Arabidopsis*, *HvACBP1/2/6/8* were classified into Class I (small ACBPs), *HvACBP3/4* into Class II (ANK-ACBPs), *HvACBP5* into Class III (large ACBPs), and *HvACBP7* into Class IV (Kelch-ACBPs).

Collinearity analysis revealed the presence of collinear blocks in the barley and *Arabidopsis* genomes, although no collinear pairs of ACBP genes were identified. In contrast, comparisons of the barley, rice, and wheat genomes revealed four pairs of collinear ACBP genes (Fig. 1b).

### Gene structure, conserved motif, and domain analysis

**Fig. 2:**
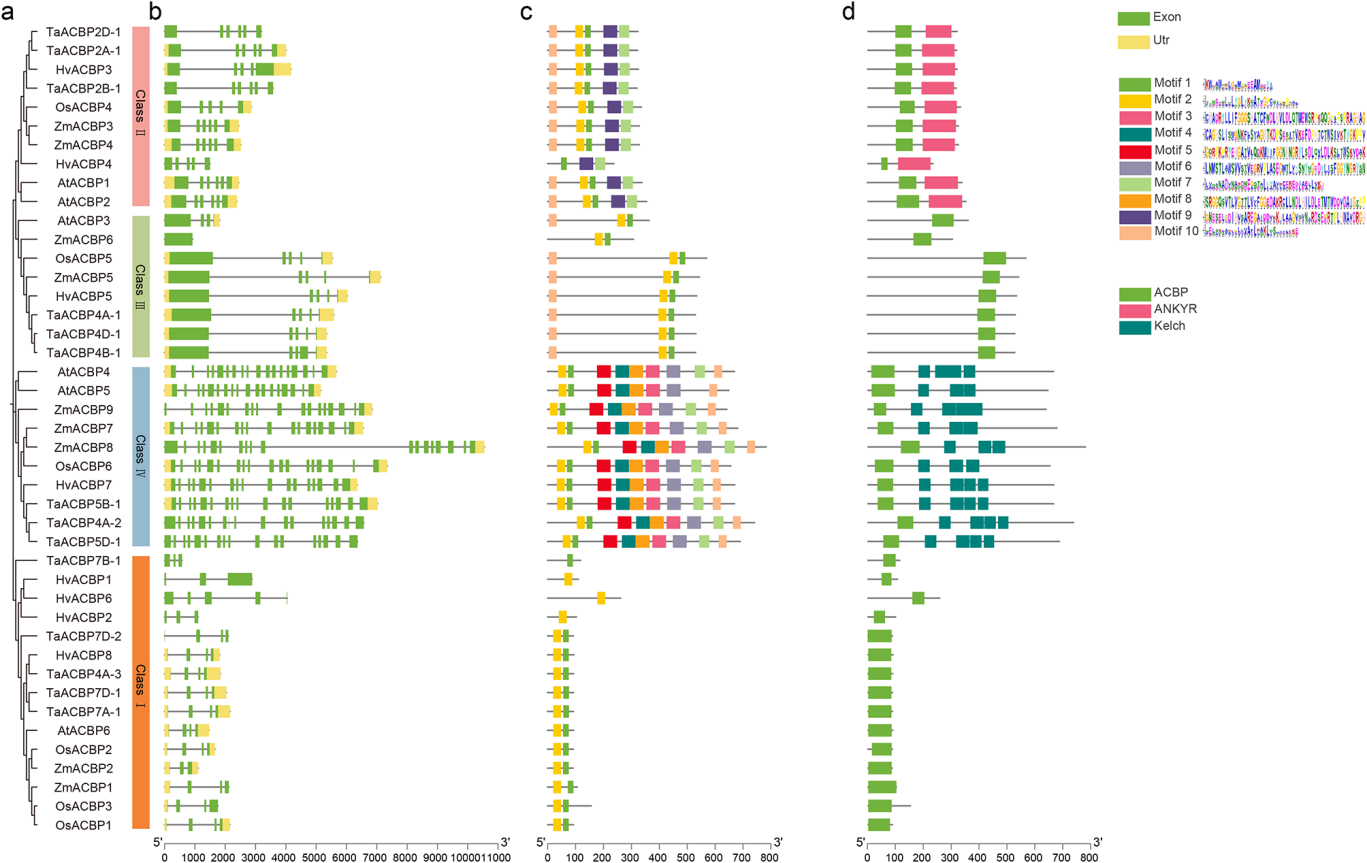
Maximum likelihood phylogenetic structure and motifs of acyl-CoA-binding protein (ACBP) genes in *Hordeum vulgare* L. (**a**) Phylogenetic tree and *HvACBP* subfamilies, further divided into four groups. (**b**) HvACBP exon–intron organization. (**c**) ACBP motifs. (**d**) Ankyrin domains are conserved in the ANK-ACBP subfamily and the Kelch domain is conserved in the Kelch-ACBP subfamily

Gene structure analysis revealed that most of the genes contained upstream regulatory regions, and all contained exons, introns, and a conserved ACBP domain (Fig. 2a, b and d), with similar exon numbers and positions. The ACBP protein motifs and conserved domains were examined in *Arabidopsis*, rice, wheat, maize, and barley. The structural motifs of ACBPs proteins contained ten protein motifs (Motifs 1–10) of 0.21–50 amino acids in length. Motif 1 and Motif 2 are highly conserved in the ACBP protein family. Motif 9 was identified only in Class II, and Motifs 3–6 and 8 were only identified in Class IV (Fig. 2c). All ACBP proteins contained an ACBP domain. All ANK-ACBPs contained an ANKYR domain at the C-terminus, and all Kelch-ACBPs had an unequal number of Kelch domains.

### HvACBP protein properties, secondary and tertiary structure

We analyzed the physical and chemical properties of HvACBP proteins. Their sizes ranged from 93 (HvACBP8) to 669 amino acids (HvACBP7), with molecular weights of 10.24 to 72.62 kDa (Table 1). The theoretical isoelectric point ranged from 4.23 (HvACBP5) to 6.22 (HvACBP1). Their instability index ranged from 30.54 (HvACBP2) to 52.97 (HvACBP3); those of HvACBP1, HvACBP3, HvACBP4, HvACBP5, and HvACBP7 exceeded 40, indicating that these proteins are unstable. Aliphatic index ranged from 66.33 (HvACBP1) to 84.07 (HvACBP5), suggesting relatively consistent thermal stability. The GRAVY (Grand average of hydropathicity) were negative, indicating that they are hydrophilic proteins (positive values indicate that proteins are more inclined towards hydrophobicity, whereas negative values indicate that proteins are more inclined towards hydrophilicity). The proteins were all predicted to localize in the cytoplasm.

**Table 1:** Properties of HvACBP proteins

Class	Number	Gene name	Number of amino acids	Theoretical pI	Molecular weight (kDa)	Instability index	Aliphatic index	Grand average of hydropathicity	Alpha helix (%)	Extended strand (%)	Beta turn (%)	Random coil (%)	Domain	Euk-mPLoc 2.0
Class I	HvACBP1	*HORVU.MOREX.r3.1HG0076680.1*	109	6.22	12.13	41.08	66.33	-0.189	0.3486	0.2661	0.0367	0.3486	ACBP	Cytoplasm
	HvACBP2	*HORVU.MOREX.r3.2HG0124500.1*	102	5.28	10.98	30.54	69.8	-0.089	0.2745	0.2745	0.1275	0.3235	ACBP	Cytoplasm
	HvACBP6	*HORVU.MOREX.r3.4HG0409130.1*	260	6.04	28.95	38.41	74.27	-0.369	0.3231	0.2154	0.0769	0.3846	ACBP	Cytoplasm
	HvACBP8	*HORVU.MOREX.r3.7HG0640790.1*	93	4.99	10.24	35.08	69.46	-0.566	0.6774	0.0215	0.0323	0.2688	ACBP	Cytoplasm
Class II	HvACBP3	*HORVU.MOREX.r3.2HG0213220.2*	324	4.4	34.61	52.97	78.43	-0.351	0.4414	0.0586	0.0802	0.4198	ACBP	Cytoplasm
	HvACBP4	*HORVU.MOREX.r3.2HG0213280.1*	236	4.6	26.19	42.2	83.47	-0.226	0.4195	0.1102	0.1017	0.3686	ACBP	Cytoplasm
Class III	HvACBP5	*HORVU.MOREX.r3.4HG0392690.1*	533	4.23	56.99	48.9	84.07	-0.417	0.5291	0.0657	0.0732	0.3321	ACBP	Cytoplasm
Class IV	HvACBP7	*HORVU.MOREX.r3.5HG0530170.1*	669	5	72.62	46.26	78.61	-0.418	0.3572	0.1659	0.0673	0.4096	ACBP	Cytoplasm

**Fig. 3:**
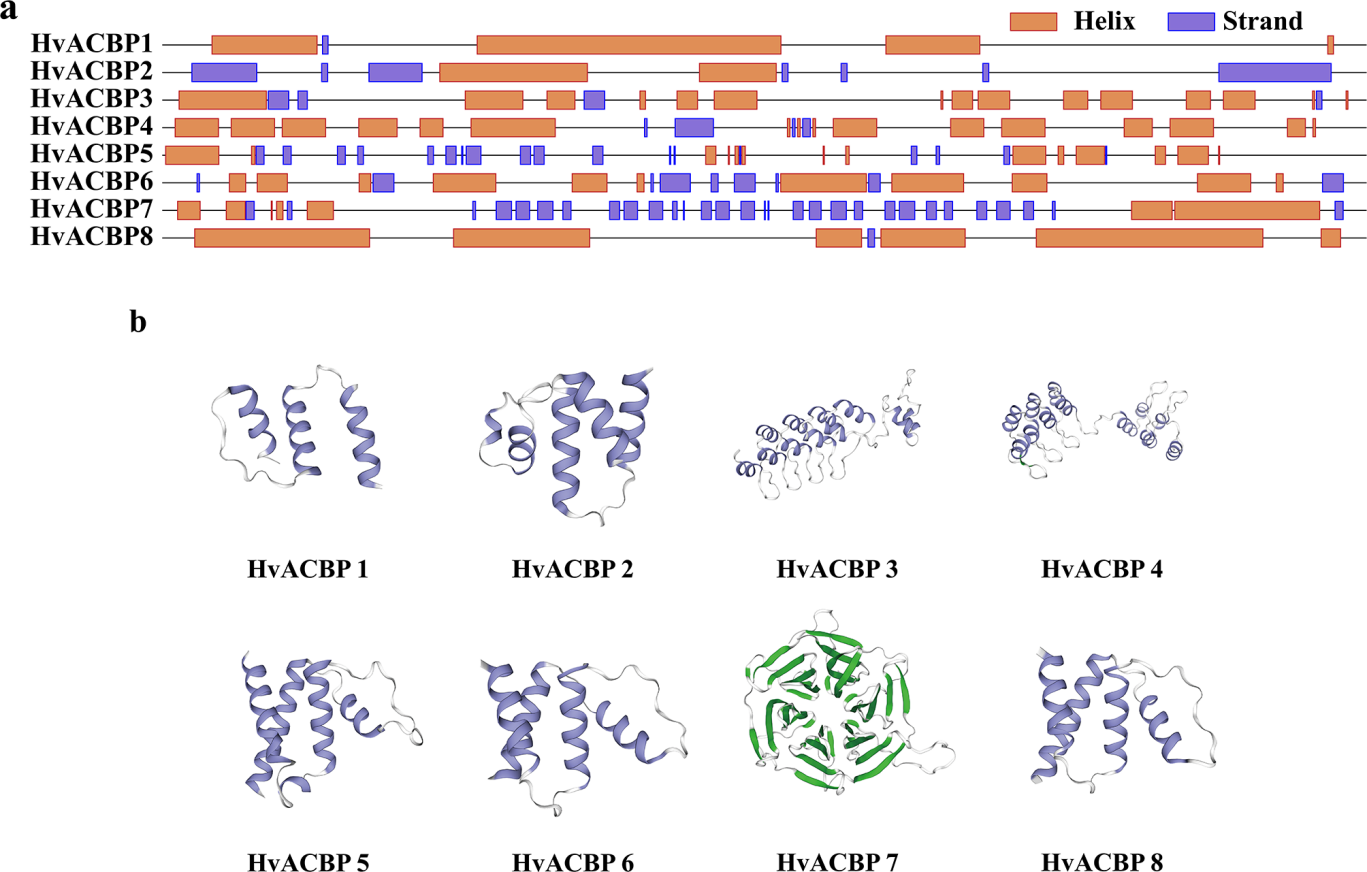
Predicted secondary and tertiary structure of acyl-CoA-binding proteins (ACBPs) in *Hordeum vulgare* L. (**a**) HvACBP secondary structure. Orange block: α-helical conformation. Blue: random- coil structure. (**b**) HvACBP tertiary structure. Blue block: α-helical conformation. Green: β-fold conformation. White line: random-coil structure

Predictive analysis to predict HvACBP secondary and tertiary structures prediction revealed notable differences among the four gene Classes (Fig. 3). Class I ACBP proteins contained a structural motif characterized by 3–5 α-helical segments, accounting for 32.31–67.74% of the overall amino acid content (Table 1; Fig. 3b). HvACBP5 showed a similar structure to that of Class I. Class II HvACBPs exhibited more α-helical than random coil structures in terms of their total amino-acid composition. In contrast, for Class IV, most of the amino acids exhibited a random coil conformation. The Class IV tertiary structure showed substantial segments of β-fold conformations because of the prevalence of Kelch structures.

### Cis-element analysis of HvACBP promoters

Cis-elements within eight HvACBP gene promoters were classified into three categories. The first category included elements closely associated with phytohormones, including auxins, MeJA, salicylic acid, ABA, gibberellin, and ethylene. The second category included elements closely associated with responses to environmental stressors, including drought, cold, wounding, anaerobic conditions, other stressors, and with defense, The third category included elements associated with plant growth and development, including meristem-specific and seed-specific expression, zein metabolism regulation, and transcription factor binding sites (specifically *MYB* and *MYC* transcription factors).

**Fig. 4:**
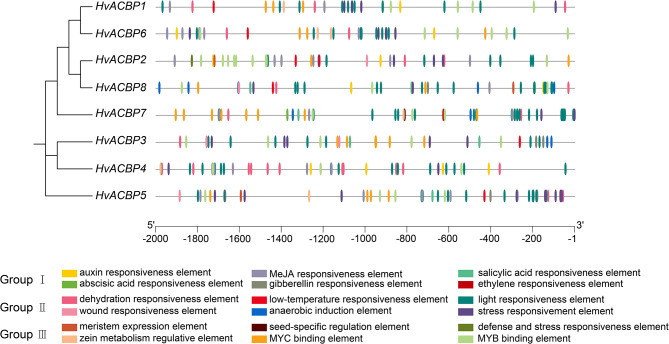
Maximum likelihood phylogenetic trees and prediction of cis-acting elements in the acyl-CoA-binding protein gene (*ACBP*) promoters of *Hordeum vulgare* L. Two-kilobase pair promoter sequences were used to analyze hormone-related cis-elements, plant growth and development-related cis-elements, and stress-related elements. Different types of cis-elements are indicated by different colored symbols adjacent to their respective promoter

All eight *HvACBP* promoters contained elements associated with drought, light regulation, MYB binding, and stress responses; these elements participate in hormonal signaling, stress responses, and transcription factor binding. Our findings strongly suggest that *HvACBPs* influence hormonal signaling pathways and environmental responses, and regulate plant growth and development (Fig. 4).

### Tissue-specific expression of *HvACBPs*

*HvACBPs* showed tissue-specific expression. *HvACBP6* expression was negligible in all the examined tissue types. *HvACBP2* expression was moderate in the ROO2, EPI, SEN, and EMB tissue types. *HvACBP1* was expressed predominantly during grain development, with minimal expression observed in the other tissue types. *HvACBP4* was few expressed in all detected tissues. Expression of the other four *HvACBPs* varied significantly within tissues.

**Fig. 5:**
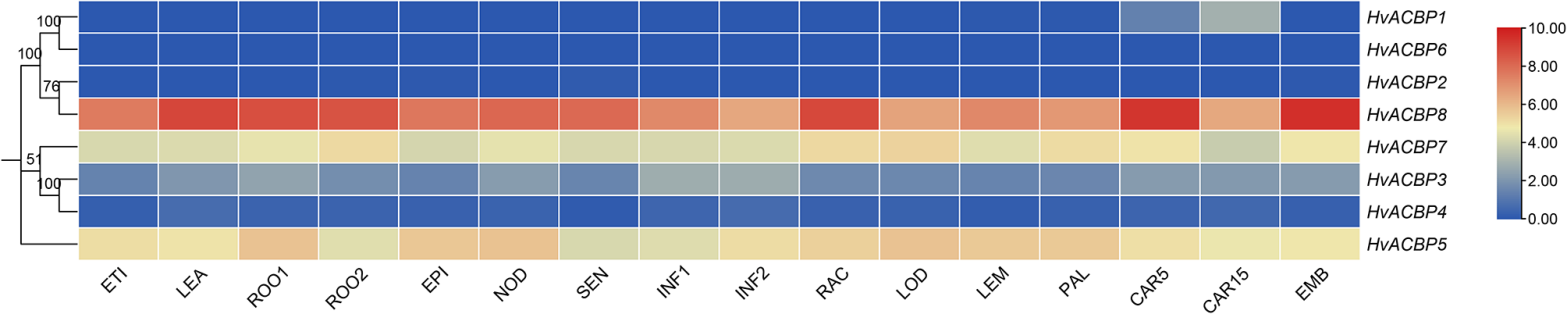
Relative expression of acyl-CoA-binding protein genes (ACBPs) in different tissues of Hordeum vulgare L. Heat maps reflect the fragments per kilobase of transcript per million mapped fragments (FPKM) of *HvACBPs*. The color gradient from red to blue indicates high to low expression. ETI: seedlings were grown to 10 days after planting (dap) in the dark to isolate etiolated leaves. LEA: leaf tissue, 17 dap. ROO1: root tissue, 17 dap. ROO2: root tissue, 28 dap. EPI: epidermal strips were obtained from plant leaf tissue at 28 dap. NOD: third stem internode, 42 dap. SEN: senescing leaf, 56 dap. INF1: whole developing inflorescence tissue was sampled at 30 dap. INF2: whole developing inflorescence tissue, 50 dap. RAC: rachis, 35 dap. LOD: lodicule, 42 dap. LEM: lemma. PAL: palea. CAR5: development grain, 5 days post-anthesis(dpa). CAR15: development grain, 15 dpa. EMB: embryonic tissue, 4 d after germination of mature grain in Petri plates in the dark in the laboratory

*HvACBP8* exhibited the highest expression across all tissue types, particularly in the young root, shoot, and grain tissues (LEA, ROO1, CAR5, and EMB). In all tissue types, *HvACBP3* and *HvACBP4* (in the ANK-ACBP subfamily) exhibited similar expression patterns, although *HvACBP3* expression substantially higher than that of *HvACBP4*, suggesting its importance in barley. Classes III and IV each included one member (*HvACBP5* and *HvACBP7*, respectively) that was abundantly expressed across all tissue types. The robust expression of *HvACBP3/4/5/7/8* across all barley tissues throughout the growth and developmental phases indicated the conservation and pivotal roles of *ACBP* genes in physiological processes in barley (Fig. 5).

### *HvACBP* responses to hormonal stress induction

**Fig. 6:**
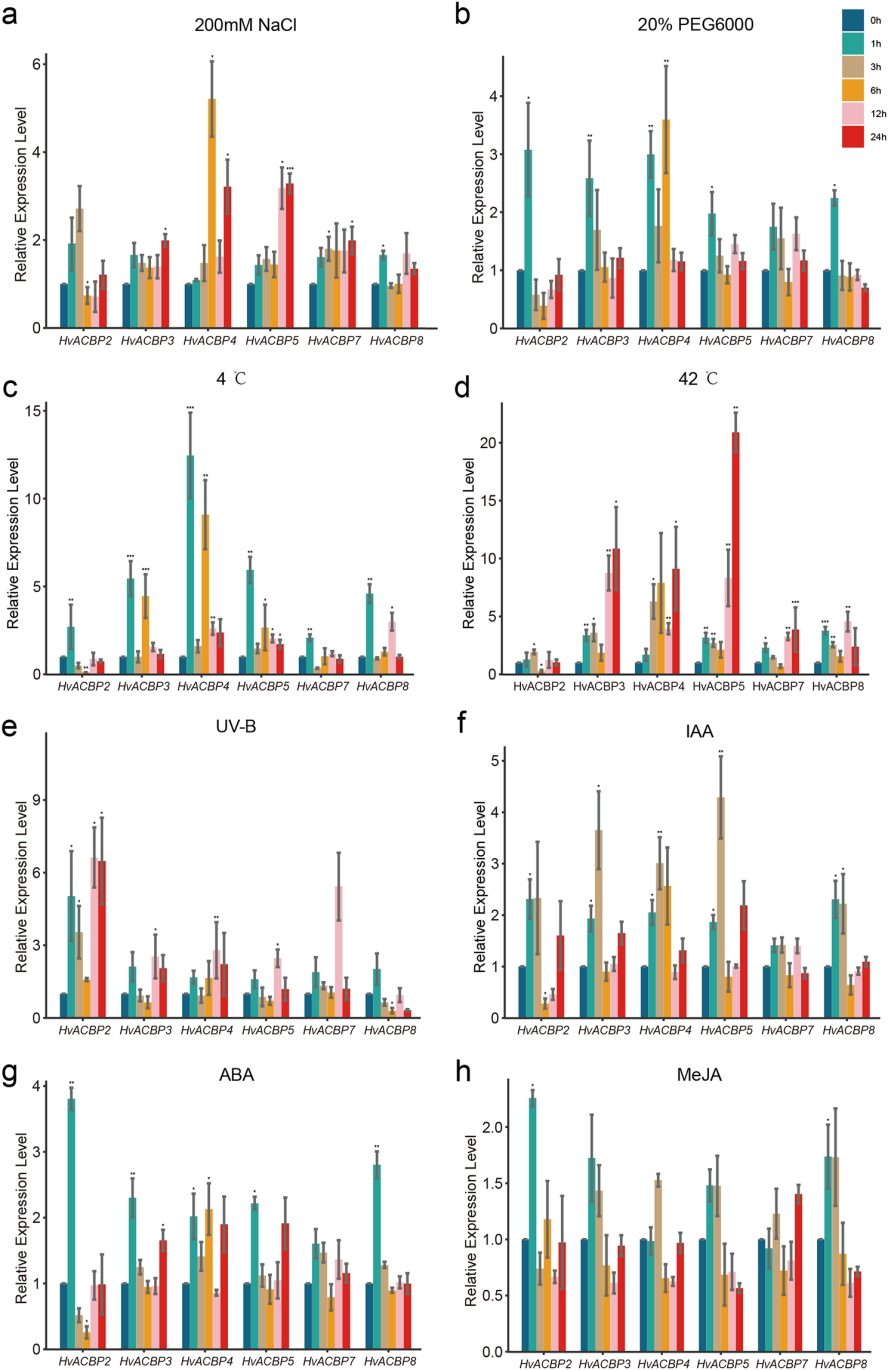
Acyl-CoA-binding protein gene (*ACBP*) expression in *Hordeum vulgare* L. under hormone treatment and abiotic stress.Stress was induced by hormone treatment with indoel-3-acetic acid (IAA) at 0.15 µmol/L or abscisic acid (ABA) at 100 µmol/L or methyl jasmonate (MeJA) at 100 µmol/L, and *ACBP* expression was measured after 1, 3, 6, 12, and 24 h. Responses to abiotic stressors were measured 1, 3, 6, 12, and 24 h after treatment with 200 mM NaCl, 20% polyethylene glycol (PEG6000), or 30 µW/cm² UV radiation (growth lamp was turned off after 12 h of UV treatment, and no UV treatment was provided after 12 h). Cold and heat stress were applied via treatment at 4 °C and 42 °C, respectively. The expression levels of genes in the control were defined as “1”. The values are presented as the means of three replicates. “*” as significant at *P* ≤ 0.05, “**” as significant at *P* ≤ 0.01, “***” as significant at *P* ≤ 0.001

Bioinformatics analysis of the promoter elements revealed the presence of cis-regulatory elements for IAA, ABA, and MeJA in the upstream region of *HvACBP* (Fig. 4). The expression of *HvACBP1/6* was not detected. Treatment of barley seedlings with IAA, ABA, and MeJA induced *HvACBP* expression to varying degrees (Fig. 6f-h). Following IAA treatment, all genes, except for *HvACBP7*, exhibited robust upregulation at 1 and 3 h post-induction. However, at 6 h post-induction, *HvACBP* expression began to decline toward (or below) basal levels, ultimately returning to their baseline expression at 12–24 h post-induction (Fig. 6f).

Compared with the effects of ABA, MeJA caused longer and more sustained induction of *HvACBPs* (Fig. 6g, h). At 3 h post-treatment, the expression of *HvACBPs* was still elevated, although it had declined at 6, 12, and 24 h. In contrast, at 3 h post-ABA induction, *HvACBP* expression had reverted to pre-induction levels.

### *HvACBP* expression under abiotic stress

We examined the regulation of *HvACBP* expression following exposure to abiotic stressors (salinity, drought, temperature variation, and UV radiation) (Fig. 6). *HvACBP1* and *HvACBP6* expression was not detected. Under salt stress, six of the *HvACBP* genes exhibited varying degrees of upregulation; in contrast, at 6 h, *HvACBP2* was slightly downregulated (Fig. 6a). *HvACBP4* exhibited significant upregulation (5.2-fold) after 6 h of salt stress, whereas *HvACBP3* exhibited a moderate response. HvACBP5 exhibited significant upregulation after 12 h of salt stress. Six *HvACBPs* exhibited rapid upregulation within 1 h of drought induction (Fig. 6b), although their expression declined over time. *HvACBP2* expression was lower than that at baseline after 6 and 12 h of drought stress.

*HvACBP* expression was differentially regulated under high and low temperature stress (Fig. 6c, d). Following exposure to 4 °C, *HvACBP* expression was rapidly upregulated, with *HvACBP4* exhibiting a remarkable 12-fold increase after 1 h. Heat stress induced significant upregulation of most of the *HvACBP*s, particulary for *HvACBP5*, which exhibited 20-fold upregulation after 24 h.

Six of the *HvACBPs* were differentially regulated in response to UV radiation, with upregulation at 1 h post-exposure (Fig. 6e), followed by a decline and subsequent increase to a peak at 12 h. *HvACBP2* and *HvACBP7* showed the most significant responses to UV stress, with upregulation of 6.6- and 5.4-fold, respectively. These findings suggest that *HvACBPs* are crucial in mediating abiotic stress-tolerance responses.

### Haplotype variations analysis of *HvACBP7* and *HvACBP8* between barley and Tibetan barley

**Fig. 7:**
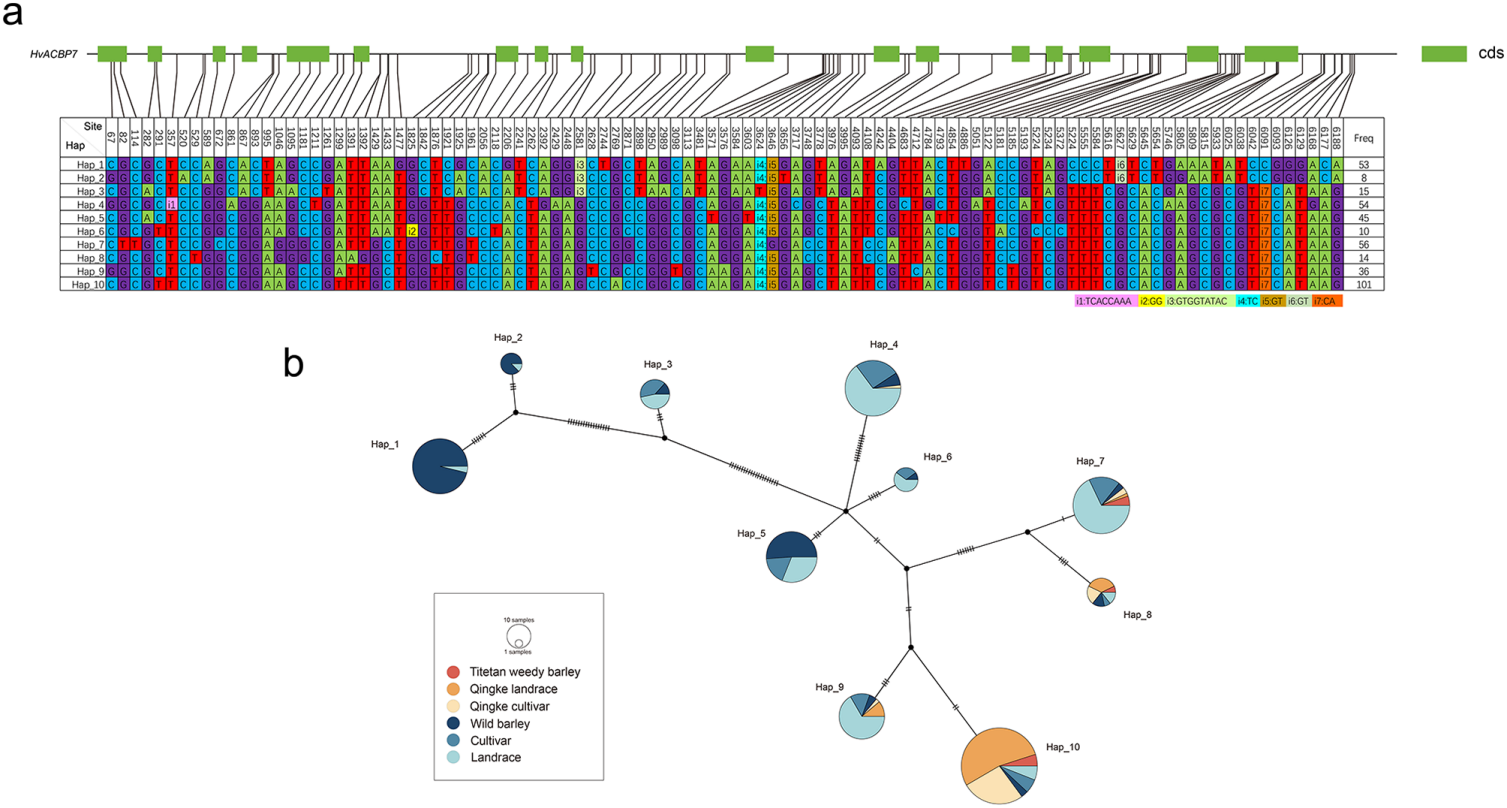
*HvACBP7* haplotype analysis. (**a**) Single-nucleotide polymorphisms (SNPs) were identified for haplotype analysis. Light-green rectangles: exons; straight lines: introns; SNPs in the promoter and coding sequence are shown in the upper table. Indels are represented by i1–7. (**b**) Proportions of each barley resource having each haplotype, with haplotype network analysis

To investigate the role of the ACBP genes in environmental adaptation in barley, we analyzed the haplotypes of ACBP members showing high expression levels. Ten haplotypes were identified in *HvACBP7*. Among them, Hap_10 was the most prevalent, present in 101 samples, whereas Hap_2 was the rarest, present in only 8 (Fig. 7; Table 2). Wild barley predominantly exhibited Hap_5 (23.7%) and Hap_1 (52.6%), whereas nearly all Tibetan barley samples exhibited Hap_7–10 (mostly HAP_10, 80.4%). Haplotype network analysis revealed a distant genetic relationship between Hap_7–10 and Hap_1–2, indicating that *HvACBP7* gathered in some new haplotypes during the domestication of Tibetan barley. Protein sequences analysis (Additional Data_2) revealed a substitution event (replacement of tyrosine with phenylalanine) in Hap_10 at amino acid 199 of HvACBP7.

**Table 2:** Haploid frequency distribution data

Haplotype	Titetan_weedy_barley	Qingke_landrace_barley	Qingke_cultivar_barley	Wild_barley	Cultivar_barley	Landrace_barley
Hap_1	0	0	0	51	0	2
Hap_2	0	0	0	7	0	1
Hap_3	0	0	0	2	6	7
Hap_4	0	0	1	4	14	35
Hap_5	0	0	0	23	8	14
Hap_6	0	0	0	1	3	6
Hap_7	3	1	2	2	10	38
Hap_8	1	5	3	2	1	2
Hap_9	0	4	1	2	5	24
Hap_10	5	54	27	3	6	6

Nine haplotypes were identified in *HvACBP8* (Supplementary Data 3). Hap_9, Hap_8, and Hap_6 were the dominate haplotypes. Among them, Tibetan barley mainly contained Hap_6, Hap7, Hap_8, and Hap_9. Remarkably, wild barley had a higher distribution proportion of these haplotypes. Protein sequence analysis revealed no amino acid variations between haplotypes, suggesting that the function of HvACBP8 is highly conserved in function.

## Discussion

### ACBPs are structurally conserved and colinear in gramineous crops

The functions of ACBPs have been extensively studied in higher plants, including *Arabidopsis*, rice [27], wheat [40], rapeseed [41], maize [42], and soybeans [43], but not in barley. Here, we comprehensively characterized *ACBP* sequences in barley, by comparing them with those of *Arabidopsis*, rice, maize, wheat, soybeans, and sorghum. All taxa were contained all four *ACBP* subfamilies, which shared a common ACBP structural domain and had a similar exon number and position, consistent with prior findings for maize [42]. This finding suggests that *ACBP* was conserved during plant evolution. Within subfamilies, different plants contain varying numbers of members, which may be related to genome size and gene copy number during evolution, such as the tandem repeats of *HvACBP3* and *HvACBP4*.

In addition, although the four subfamilies were conserved, and the collinearity of *ACBP* between the dicotyledonous plants, *Arabidopsis* and soybean, and barley was lower than that between monocotyledonous plants. This result suggests that, although homologous *ACBPs* with identical functional structural domains are common across plant species and that the four subfamilies evolved before the divergence of monocotyledonous and dicotyledonous plants, *ACBP* sequences widely varied during plant diversification, further indicating their importance and conserved roles, as well as highlighting the functional divergence among HvACBPs.

### ACBP regulates responses to exogenous hormones and modulates plant growth and development

*ACBPs* perform critical roles in plant growth and development, as indicated by their tissue-specific expression and involvement in diverse phytohormone pathways [13, 44].

Previous studies revealed functional redundancy among *ACBP* members. For example, *AtACBP1* and *AtACBP2* have similar expression patterns, with the loss of one gene leading to compensatory expression of the other gene. The double mutant of *atacbp1* and *atacbp2* can cause embryonic death in seeds [45]. *HvACBP3* and *HvACBP4*, which are tandem repeat genes, show similar expression patterns, suggesting their conserved and redundant functions in regulating seed development. These similar expression profiles suggest that *HvACBPs* are involved in barley growth and development. For example, the small ACBP, *AtACBP6*, of *Arabidopsis* is highly expressed in pollen, microspores, and trichome cells [14], whereas *OsACBP1/2* in rice is highly expressed in developing grains [13, 28]. The variation in AtACBP6 affects lipid accumulation in Arabidopsis seeds [30]. The specific high expression of *HvACBP1/8* in grains suggests their function in regulating seed development.

Analysis of cis-elements indicated that *HvACBPs* are involved in the cross-talk of phytohormones, and the effects of hormone treatment also confirmed that *HvACBPs* are involved in the plant hormone response, which is crucial for various aspects of plant growth, development, and stress responses, these results highlighting the potential regulatory functions of *HvACBPs* in barley growth, development, and environmental adaptation.

### *ACBPs* show functional conservation and sub-functionalization in paralogs in responses to abiotic stress

ACBPs are vital in plant responses to various stressors. In *Arabidopsis*, overexpression of *AtACBP1* enhances the sensitivity to salt stress by activating ABA expression via *AtAREB1* [46]. In soybeans, salt tolerance is mediated by *GmACBP3* and *GmACBP4* (Class II) via alternative splicing [39]. The expression level of the *OsACBP4* gene in rice rapidly increases under high-salt treatment. In barley, *HvACBP4* responds strongly to salt stress, suggesting a role for this gene salt tolerance, whereas *HvACBP3* reacts more slowly, indicating distinct roles for these duplicated genes. In *Arabidopsis*, *AtACBP2* is closely associated with drought response via regulation of ABA-dependent guard-cell closure to improve drought resistance [47–50]. *AtACBP3* and *AtACBP4* are associated with drought responses [51–53]. *OsACBP4* and *OsACBP5* in rice; as well as *GhACBP1*, *GhACBP3*, and *GhACBP6* in cotton, are induced by drought [27, 54, 55]. In barley, the six detected *HvACBPs* upregulated under salt treatment, suggesting that they are related to drought tolerance in barley. *AtACBP6* overexpression enhances stability under cold stress, whereas *AtACBP1* knockout enhances freezing resistance in *Arabidopsis* [29, 56]. Under cold stress, all six *OsACBP*s were downregulated [27]. In cotton, *GhACBP1*, *GhACBP3*, and *GhACBP6* respond to low-temperature stress, whereas *GhACBP6* exhibits significant regulation under heat stress [54]. We found that the *HvACBPs* exhibited varied responses to heat and cold but responded more strongly to heat than to cold. Therefore, *HvACBPs* respond rapidly and in diverse manners to temperature stress, highlighting their importance in temperature adaptation in barley. UV radiation induces membrane damage [57]. Our findings reveal that *ACBPs* participate in repairing UV damage in barley.

The ACBP gene shows sub-functionalization in paralogs. Among Ankry-ACBP subfamily genes, in *Arabidopsis, AtACBP1* is only expressed in the trichomes, whereas *AtACBP2* is only expressed in guard cells, indicating that *AtACBP2* is associated with stomatal opening and the drought response [21, 22, 52, 47]. In barley, *HvACBP3* and *HvACBP4* are tandem repeat genes; *HvACBP3* expression was higher during growth and breeding, whereas *HvACBP4* is more strongly responsive to abiotic stress. *HvACBP2*, a small ACBP in barley, showed greater sensitivity to UV-B than did *HvACBP8*. These results indicate that ACBPs underwent sub-functionalization in paralogs during species evolution.

The Tibetan Plateau, the predominant cultivation region for Tibetan barley, which was domesticated from eastern domesticated barley, is characterized by harsh environmental conditions, including low temperatures, substantial diurnal temperature fluctuations, low latitudes, high elevations, low-density air, intense solar radiation, and high UV-B exposure [48]. ACBPs constitute a well-conserved family of proteins in eukaryotes that are important in stress responses and development [49]. The chromosomal region containing *HvACBP7* was under selective pressure during the domestication of barley in the Tibetan region [50]. Analysis of *HvACBP7* and *HvACBP8* haplotypes showed that during the domestication of barley, *HvACBP7/8* were selected to adapt to the unique environment of the Tibetan Plateau, but their amino acid sequences remained conserved, suggesting the importance and conservation of *ACBPs* in the growth, development, and environmental adaptation of barley; however, the variation of the cis-elements in the promoter requires further analysis.

## Conclusions

*HvACBPs* respond to multiple stressors, underscoring their crucial role in stress resilience in barley, as well as revealing the importance of ACBPs in plant growth, development, and stress adaptation, particularly abiotic stress. Our results provide a foundation for the further functional analysis of *HvACBP* and theoretical basis for improving barley via breeding. We determined mechanisms of plant ecological adaptation in extreme environments.

## Materials and methods

### Plant material, growth, and abiotic stress conditions

Barley seeds (*H. vulgare* L., Morex) were provided by the Chinese Academy of Agricultural Sciences. The seeds were disinfected with 0.1% NaClO and 75% ethanol, rinsed with distilled water, and placed on moist filter paper into dark for 24 h. The germinated seeds were transferred to hydroponic containers and grown under 24 °C with a 14 h light/10 h dark cycle. The nutrient solution (1/2 Hoagland solution) was replaced every 3 d. Different treatments were applied after 4 weeks.

The 4-week-old seedlings were subjected low-temperature stress (4 °C), high-temperature stress (42 °C), 200 mM NaCl application, 20% polyethylene glycol (PEG 6000) treatment, UV radiation at 30 µW/cm2, 0.15 µmol/L IAA treatment, 100 µmol/L ABA treatment, and 100 µmol/L MeJA treatment. The seedlings were harvested at 0, 1, 3, 6, 12, and 24 h post-treatment, immediately frozen in liquid nitrogen, and stored at − 80 °C for subsequent experiments.

### Sequence retrieval and identification of *ACBPs*

We performed a BLASTP search using the *ACBP* family from *Arabidopsis* (https://www.arabidopsis.org/) in the barley genome database (http://barlex.barleysequence.org/) with an e-value of 10^− 10^. We also downloaded the ACBP domain file (PF00887) from the Pfam website (http://pfam.xfam.org/) and uploaded it to the HMM website (https://www.ebi.ac.uk/Tools/hmmer/search/hmmsearch) to search for genes in the barley genome potentially containing this conserved domain. Duplicate genes were removed by comparing the results of the two different searches. The remaining genes were validated for the presence of the ACBP-type domain using the CD search (https://www.ncbi.nlm.nih.gov/cdd/) and SMART (http://smart.embl-heidelberg.de/) websites.

### *HvACBP* structure and conserved motif analysis

The structures of the *HvACBPs* were deduced from the coding and genomic sequences using a gene structure displayer (https://gsds.gao-lab.org/). Conserved motifs in each HvACBP were analyzed using MEME Suite (https://meme-suite.org/meme/tools/meme).

### Analysis of cis-acting elements in *HvACBP* promoters

A region approximately 2000 bp upstream of the start codon (ATG) was investigated from the reference genome sequence of MorexV3, Cis-acting elements were predicted using the PlantCare website (http://bioinformatics.psb.ugent.be/webtools/plantcare/html/).

### Expression patterns of *HvACBP* family in different tissues

*HvACBPs* transcriptome data (fragments per kilobase of transcript per million mapped fragments) were downloaded from the barley genome database (http://barlex.barleysequence.org/). Transcriptome data were normalized by logarithms, and the expression heat maps were drawn using R4.2.1 software.

### Phylogenetic analysis of *HvACBPs*

To calculate the correlations between *HvACBPs* and their counterparts in various species, the ACBP protein sequences of Arabidopsis, rice, maize, soybean, sorghum, wheat, and barley were subjected to multiple sequence alignment using the Clustal W tool. A phylogenetic tree was constructed using the maximum likelihood (ML) method via MEGA 7, employing the WAG + G model. The resultant phylogenetic tree was further refined and visualized using iTOL v6 (https://itol.embl.de/).

### Protein physical and chemical properties and prediction of secondary and tertiary structures

We used ExPASy (https://web.expasy.org/protparam/) to compute the amino acid content, isoelectric point (pI), molecular weight (average), instability coefficient, aliphatic index, and average hydrophobicity index. The SOPMA (https://npsa-prabi.ibcp.fr/cgi-bin/npsa_automat.pl?page=npsa_sopma.html) and PredictProtein (https://predictprotein.org/) websites were used to predict the secondary structure of the proteins. The tertiary structure of HvACBP proteins was predicted using the SWISS-MODEL Interactive Workshop (https://www.swissmodel.expasy.org/interactive).

### RNA isolation, cDNA synthesis, and quantitative real-time PCR analysis

Total RNA extraction was performed using the FastPure Universal Plant Total RNA Isolation Kit (Vazyme, Nanjing, China). RNA was reverse-transcribed into cDNA using the HiScript III RT SuperMix for qPCR (+ gDNA wiper) kit (Vazyme, Nanjing, China). Quantitative real-time PCR (qRT-PCR) was conducted using Taq Pro Universal SYBR qPCR Master Mix (Vazyme, Nanjing, China). The internal reference gene used for normalization was *HvGAPDH* (*HORVU.MOREX.r3.7HG0703580*). Each sample contained 10 µL of a reaction mixture composed of 5 µL of 2×PCR mix, 1 µL of primer mixture (10 µmol L-1 each upstream and downstream primers), 1 µL of cDNA, and 3 µL of deionized water. The PCR thermal cycling conditions were as follows: initial denaturation at 95 °C for 30 s followed by 35 cycles of denaturation at 95 °C for 5 s, annealing at 60 °C for 30 s, and extension at 95 °C for 15 s. A final extension step was performed at 60 °C for 60 s, followed by melt curve analysis at 95 °C for 15 s. The experiment was conducted in triplicate. The primers are listed in Supplementary Data.

### Haplotype analysis of HvACBP7 and HvACBP8

All single-nucleotide polymorphisms and insertions–deletions used for haplotype analysis were obtained by resequencing of Tibet barley [55]. Haplotype identification was performed using DnaSP_V5 and haplotype network analysis was performed using Arlequin.

### Electronic supplementary material

Below is the link to the electronic supplementary material.

Supplementary Material 1

Supplementary Material 2

Supplementary Material 3

Supplementary Material 4

Supplementary Material 5

## Data Availability

All datasets supporting the results of this study are included within the article and its supplementary information.

## References

[1] Yu L, Zhou C, Fan J, Shanklin J, Xu C (2021). Mechanisms and functions of membrane lipid remodeling in plants. Plant J.

[2] Hsiao AS, Yeung EC, Ye ZW, Chye ML (2015). The Arabidopsis Cytosolic Acyl-CoA-Binding proteins play combinatory roles in Pollen Development. Plant Cell Physiol.

[3] Li HY, Xiao S, Chye ML (2008). Ethylene- and pathogen-inducible Arabidopsis acyl-CoA-binding protein 4 interacts with an ethylene-responsive element binding protein. J Exp Bot.

[4] Xu C, Shanklin J (2016). Triacylglycerol metabolism, function, and Accumulation in Plant vegetative tissues. Annu Rev Plant Biol.

[5] Noack LC, Jaillais Y (2020). Functions of anionic lipids in plants. Annu Rev Plant Biol.

[6] Lim GH, Singhal R, Kachroo A, Kachroo P (2017). Fatty acid– and lipid-mediated signaling in Plant Defense. Annu Rev Plant Biol.

[7] Li-Beisson Y, Shorrosh B, Beisson F, Andersson MX, Arondel V, Bates PD, Baud S, Bird D, DeBono A, Durrett TP et al. Acyl-Lipid Metabolism Arabidopsis Book. 2013. p. 11. 10.1199/tab.0161 PMC3563272 23505340

[8] Lung SC, Chye ML (2016). The binding versatility of plant acyl-CoA-binding proteins and their significance in lipid metabolism. Biochimica et Biophysica Acta (BBA) - Mol. Cell Biol Lipids.

[9] Nie Z, Wang Y, Wu C, Li Y, Kang G, Qin H, Zeng R (2018). Acyl-CoA-binding protein family members in laticifers are possibly involved in lipid and latex metabolism of Hevea brasiliensis (the para rubber tree). BMC Genomics.

[10] Raboanatahiry N, Wang B, Yu L, Li M (2018). Functional and Structural Diversity of Acyl-Coa Binding Proteins in oil crops. Front Genet.

[11] Leung KC, Li HY, Mishra G, Chye ML (2005). ACBP4 and ACBP5, novel Arabidopsis acyl-CoA-binding proteins with kelch motifs that bind oleoyl-CoA. Plant Mol Biol.

[12] Sasaki Y, Nagano Y (2004). Plant acetyl-CoA carboxylase: structure, biosynthesis, regulation, and gene manipulation for plant breeding. Biosci Biotechnol Biochem.

[13] Lai SH, Chye ML (2021). Plant Acyl-CoA-Binding proteins-their lipid and protein interactors in Abiotic and Biotic stresses. Cells.

[14] Chen QF, Xiao S, Chye ML (2008). Overexpression of the Arabidopsis 10-Kilodalton acyl-coenzyme A-Binding protein ACBP6 enhances freezing Tolerance. Plant Physiol.

[15] Meng W, Hsiao AS, Gao C, Jiang L, Chye ML (2014). Subcellular localization of rice acyl-CoA‐binding proteins (ACBPs) indicates that OsACBP6::GFP is targeted to the peroxisomes. New Phytol.

[16] Qiao K, Wang M, Takano T, Liu S (2018). Overexpression of Acyl-CoA-Binding protein 1 (ChACBP1) from saline-alkali-tolerant Chlorella sp. Enhances stress tolerance in Arabidopsis. Front. Recent dev. Plant Sci.

[17] Chye ML, Huang BQ, Zee SY (2002). Isolation of a gene encoding Arabidopsis membrane-associated acyl‐CoA binding protein and immunolocalization of its gene product. Plant J.

[18] Leung KC, Li HY, Xiao S, Tse MH, Chye ML (2005). Arabidopsis ACBP3 is an extracellularly targeted acyl-CoA-binding protein. Planta.

[19] Pastor S, Sethumadhavan K, Ullah AHJ, Gidda S, Cao H, Mason C, Chapital D, Scheffler B, Mullen R, Dyer J (2013). Molecular properties of the class III subfamily of acyl-coenyzme A binding proteins from Tung tree (Vernicia fordii). Plant Sci.

[20] Xiao S, Li HY, Zhang JP, Chan SW, Chye ML (2008). Arabidopsis acyl-CoA-binding proteins ACBP4 and ACBP5 are subcellularly localized to the cytosol and ACBP4 depletion affects membrane lipid composition. Plant Mol Biol.

[21] Du ZY, Chen MX, Chen QF, Xiao S, Chye ML (2013). Arabidopsis acyl-CoA-binding protein ACBP1 participates in the regulation of seed germination and seedling development. Plant J.

[22] Xue Y, Xiao S, Kim J, Lung SC, Chen L, Tanner JA, Suh MC, Chye M-L (2014). Arabidopsis membrane-associated acyl-CoA-binding protein ACBP1 is involved in stem cuticle formation. J Exp Bot.

[23] Zimmermann P, Hirsch-Hoffmann M, Hennig L, Gruissem W (2004). GENEVESTIGATOR. Arabidopsis Microarray Database and Analysis Toolbox. Plant Physiol.

[24] Zheng SX, Xiao S, Chye ML (2012). The gene encoding Arabidopsis acyl-CoA-binding protein 3 is pathogen inducible and subject to circadian regulation. J Exp Bot.

[25] Xiao S, Gao W, Chen QF, Chan SW, Zheng SX, Ma J, Wang M, Welti R, Chye M-L (2010). Overexpression of Arabidopsis Acyl-CoA binding protein ACBP3 promotes Starvation-Induced and Age-Dependent Leaf Senescence. Plant Cell.

[26] Xiao S, Chen QF, Chye ML (2009). Light-regulated Arabidopsis ACBP4 and ACBP5 encode cytosolic acyl-CoA-binding proteins that bind phosphatidylcholine and oleoyl-CoA ester. Plant Physiol Biochem.

[27] Meng W, Su YCF, Saunders RMK, Chye ML (2010). The rice acyl-CoA‐binding protein gene family: phylogeny, expression and functional analysis. New Phytol.

[28] Jain M, Nijhawan A, Arora R, Agarwal P, Ray S, Sharma P, Kapoor S, Tyagi AK, Khurana JP (2007). F-Box proteins in Rice. Genome-wide analysis, classification, temporal and spatial gene expression during panicle and seed development, and regulation by light and Abiotic Stress. Plant Physiol.

[29] Liao P, Chen QF, Chye ML (2014). Transgenic Arabidopsis flowers overexpressing Acyl-CoA-Binding protein ACBP6 are freezing tolerant. Plant Cell Physiol.

[30] Hsiao AS, Haslam Richard P, Michaelson Louise V, Liao P, Chen Q-F, Sooriyaarachchi S, Mowbray Sherry L, Napier Johnathan A, Tanner Julian A, Chye ML. Arabidopsis cytosolic acyl-CoA-binding proteins ACBP4, ACBP5 and ACBP6 have overlapping but distinct roles in seed development. Biosci Rep. 2014;34. 10.1042/BSR20140139 PMC4274664 25423293

[31] Guo ZH, Haslam RP, Michaelson LV, Yeung EC, Lung SC, Napier JA, Chye ML (2019). The overexpression of rice ACYL-CoA-BINDING PROTEIN2 increases grain size and bran oil content in transgenic rice. Plant J.

[32] Gao W, Xiao S, Li HY, Tsao SW, Chye ML (2008). Arabidopsis thaliana acyl-CoA-binding protein ACBP2 interacts with heavy metal binding farnesylated protein AtFP6. New Phytol.

[33] Li HY, Chye ML (2003). Membrane localization of Arabidopsis acyl-CoA binding protein ACBP2. Plant Mol Biol.

[34] Xiao S, Chye ML (2011). Overexpression of Arabidopsis ACBP3 enhances NPR1-dependent plant resistance to Pseudomonas syringe pv tomato DC3000. Plant Physiol.

[35] Du ZY, Chen MX, Chen QF, Gu JD, Chye ML (2014). Expression of Arabidopsis acyl-CoA-binding proteins AtACBP1 and AtACBP4 confers pb(II) accumulation in Brassica juncea roots. Plant Cell Environ.

[36] Panthapulakkal Narayanan S, Lung SC, Liao P, Lo C, Chye ML. The overexpression of OsACBP5 protects transgenic rice against necrotrophic, hemibiotrophic and biotrophic pathogens. Sci Rep. 2020;10. 10.1038/s41598-020-71851-9 PMC7483469 32913218

[37] Panthapulakkal Narayanan S, Liao P, Taylor PWJ, Lo C, Chye ML. Overexpression of a Monocot Acyl-CoA-Binding protein confers broad-spectrum Pathogen Protection in a Dicot. Proteomics. 2019;19. 10.1002/pmic.201800368 31054181

[38] Meng W, Xu L, Du ZY, Wang F, Zhang R, Song X, Lam SM, Shui G, Li Y, Chye ML. RICE ACYL-COA-BINDING PROTEIN6 affects Acyl-CoA homeostasis and growth in Rice. Rice. 2020;13. 10.1186/s12284-020-00435-y PMC7647982 33159253

[39] Lung SC, Lai SH, Wang H, Zhang X, Liu A, Guo ZH, Lam HM, Chye ML (2022). Oxylipin signaling in salt-stressed soybean is modulated by ligand-dependent interaction of class II acyl-CoA-binding proteins with lipoxygenase. Plant Cell.

[40] Hu P, Ren Y, Xu J, Luo W, Wang M, Song P, Guan Y, Hu H, Li C (2023). Identification of acyl-CoA-binding protein gene in Triticeae species reveals that TaACBP4A-1 and TaACBP4A-2 positively regulate powdery mildew resistance in wheat. Int J Biol Macromol.

[41] Brown AP, Johnson P, Rawsthorne S, Hills MJ (1998). Expression and properties of acyl-CoA binding protein from Brassica napus. Plant Physiol Biochem.

[42] Zhu J, Li W, Zhou Y, Pei L, Liu J, Xia X, Che R, Li H. Molecular characterization, expression and functional analysis of acyl-CoA-binding protein gene family in maize (Zea mays). BMC Plant Biol. 2021;21. 10.1186/s12870-021-02863-4 PMC7883581 33588749

[43] Ling J, Li L, Lin L, Xie H, Zheng Y, Wan X. Genome-wide identification of acyl-CoA binding proteins and possible functional prediction in legumes. Front Genet. 2023;13. 10.3389/fgene.2022.1057160 PMC9871394 36704331

[44] Xiao S, Chye ML (2011). New roles for acyl-CoA-binding proteins (ACBPs) in plant development, stress responses and lipid metabolism. Prog Lipid Res.

[45] Chen QF, Xiao S, Qi W, Mishra G, Ma J, Wang M, Chye ML (2010). The Arabidopsis acbp1acbp2 double mutant lacking acyl-CoA-binding proteins ACBP1 and ACBP2 is embryo lethal. New Phytol.

[46] Chen MX, Hu TH, Xue Y, Zhu FY, Du ZY, Lo C, Chye ML (2018). Arabidopsis Acyl-Coenzyme-A-Binding protein ACBP1 interacts with AREB1 and mediates salt and osmotic signaling in seed germination and seedling growth. Environ Exp Bot.

[47] Gao W, Li HY, Xiao S, Chye ML (2010). Acyl-CoA-binding protein 2 binds lysophospholipase 2 and lysoPC to promote tolerance to cadmium-induced oxidative stress in transgenic Arabidopsis. Plant J.

[48] Zheng D, Zhao D (2017). Characteristic of natural environment of the Tibetan Plateau. Sci Technol Rev.

[49] Hamdan MF, Lung SC, Guo ZH, Chye ML (2021). Roles of acyl-CoA-binding proteins in plant reproduction. J EXP BOT.

[50] Zeng X, Guo Y, Xu Q, Mascher M, Guo G, Li S, Mao L, Liu Q, Xia Z, Zhou J (2018). Origin and evolution of qingke barley in Tibet. Nat Commun.

[51] Saez A, Apostolova N, Gonzalez GM, Gonzalez GMP, Nicolas C, Lorenzo O, Rodriguez PL (2004). Gain of function and loss of function phenotypes of the protein phosphatase 2 C HAB1 reveal its role as a negative regulator of abscisic acid signalling. Plant J.

[52] Du ZY, Chen MX, Chen QF, Xiao S, Chye ML (2012). Overexpression of Arabidopsis acyl-CoA‐binding protein ACBP2 enhances drought tolerance. Plant Cell Environ.

[53] Kwak JM, Mori IC, Pei ZM, Leonhardt N, Torres MA, Dangl JL, Bloom RE, Bodde S, Jones JD, Schroeder JI (2003). NADPH oxidase AtrbohD and AtrbohF genes function in ROS-dependent ABA signaling in Arabidopsis. Embo J.

[54] Qin P, Shang X, Song J, Guo W (2016). Genome-wide identification of acyl-CoA-binding protein (ACBP) gene family and their functional analysis in abiotic stress tolerance in cotton. Acta Agron Sinica.

[55] Chen Y, Fu M, Li H, Wang L, Liu R, Liu Z (2023). Molecular characterization of the Acyl-CoA-Binding protein genes reveals their significant roles in Oil Accumulation and Abiotic stress response in cotton. Genes.

[56] Du ZY, Xiao S, Chen QF, Chye ML (2010). Depletion of the membrane-Associated Acyl-Coenzyme A-Binding protein ACBP1 enhances the ability of Cold Acclimation in Arabidopsis. Plant Physiol.

[57] Murphy TM (2006). Membranes as targets of ultraviolet radiation. Physiol Plant.

