# Supplementary material for: Acyl-CoA-binding protein (ACBP) genes involvement in response to abiotic stress and exogenous hormone application in barley (Hordeum vulgare L.)
Source: BMC Plant Biol. 2024 Apr 2;24:236. doi: 10.1186/s12870-024-04944-6 (PMC10985865; doi:10.1186/s12870-024-04944-6)
Supplement: Supplementary file 3 — Supplementary Material 3 [file 12870_2024_4944_MOESM3_ESM.pdf]

```
                   *        20         *        40         *        60         *        80         *       100
Hap_1  : MASSGIAYPDRFYAAAAYAGFGPGAPSAAAVSRFQNDVALLLYGLHQQATVGPCNVPKPRAWSPVEQSKWTSWHGLGSMPSAEAMRLFVKILEEEDPGWY : 100
Hap_2  : MASSGIAYPDRFYAAAAYAGFGAGAPSAAAVSRFQNDVALLLYGLHQQATVGPCNVPKPRAWSPVEQSKWTSWHGLGSMPSAEAMRLFVKILEEEDPGWY : 100
Hap_3  : MASSGIAYPDRFYAAAAYAGFGPGAPSAAAVSRFQNDVALLLYGLHQQATVGPCNVPKPRAWSPVEQSKWTSWHGLGSMPSAEAMRLFVKILEEEDPGWY : 100
Hap_4  : MASSGIAYPDRFYAAAAYAGFGAGAPSAAAVSRFQNDVALLLYGLHQQATVGPCNVPKPRAWSPVEQSKWTSWHGLGSMPSAEAMRLFVKILEEEDPGWY : 100
Hap_5  : MASSGIAYPDRFYAAAAYAGFGPGAPSAAAVSRFQNDVALLLYGLHQQATVGPCNVPKPRAWSPVEQSKWTSWHGLGSMPSAEAMRLFVKILEEEDPGWY : 100
Hap_6  : MASSGIAYPDRFYAAAAYAGFGPGAPSAAAVSRFQNDVALLLYGLHQQATVGPCNVPKPRAWSPVEQSKWTSWHGLGSMPSAEAMRLFVKILEEEDPGWY : 100
Hap_7  : MASSGIAYPDRFYAAAAYAGFGPGAPSSAAVSRFQNDVALLLYGLHQQATVGPCNVPKPRAWSPVEQSKWTSWHGLGSMPSAEAMRLFVKILEEEDPGWY : 100
Hap_8  : MASSGIAYPDRFYAAAAYAGFGPGAPSAAAVSRFQNDVALLLYGLHQQATVGPCNVPKPRAWSPVEQSKWTSWHGLGSMPSAEAMRLFVKILEEEDPGWY : 100
Hap_9  : MASSGIAYPDRFYAAAAYAGFGAGAPSAAAVSRFQNDVALLLYGLHQQATVGPCNVPKPRAWSPVEQSKWTSWHGLGSMPSAEAMRLFVKILEEEDPGWY : 100
Hap_10 : MASSGIAYPDRFYAAAAYAGFGPGAPSAAAVSRFQNDVALLLYGLHQQATVGPCNVPKPRAWSPVEQSKWTSWHGLGSMPSAEAMRLFVKILEEEDPGWY : 100
         MASSGIAYPDRFYAAAAYAGFG GAPSaAAVSRFQNDVALLLYGLHQQATVGPCNVPKPRAWSPVEQSKWTSWHGLGSMPSAEAMRLFVKILEEEDPGWY

                   *       120         *       140         *       160         *       180         *       200
Hap_1  : SRIPEFINPQPVVDIEMHKPKEEPGIVPALTNGTGTSSIPEPKTISENGSSMETQDKVVILEGLSTVSAHEEWTALSVSGQRPKPRYEHGATVLQDKMYV : 200
Hap_2  : SRIPEFINPQPVVDIEMHKPKEEPGIVPALTNGTGTSSIPEPKTISENGSSMETQDKVVILEGLSTVSAHEEWTALSVSGQRPKPRYEHGATVLQDKMYV : 200
Hap_3  : SRIPEFINPQPVVDIEMHKPKEEPGIVPALTNGTGTSSIPEPKTISENGSSMETQDKVVILEGLSTVSAHKEWTALSVSGQRPKPRYEHGATVLQDKMYV : 200
Hap_4  : SRIPEFINPQPVVDIEMHKPKEEPGIVPALTNGTGTSSIPEPKTISENGSSMETQDKVVILEGLSTVSAHEEWTALSVSGQRPKPRYEHGATVLQDKMYV : 200
Hap_5  : SRIPEFINPQPVVDIEMHKPKEEPGIVPALTNGTGTSSIPEPKTISENGSSMETQDKVVILEGLSTVSAHEEWTALSVSGQRPKPRYEHGATVLQDKMYV : 200
Hap_6  : SRIPEFINPQPVVDIEMHKPKEEPGIVPALTNGTGTSSIPEPKTISENGSSMETQDKVVILEGLSTVSAHEEWTALSVSGQRPKPRYEHGATVLQDKMYV : 200
Hap_7  : SRIPEFINPQPVVDIEMHKPKEEPGIVPALTNGTGTSSIPEPKTISENGSSMETQDKVVILEGLSTVSAHEEWTALSVSGQRPKPRYEHGATVLQDKMYV : 200
Hap_8  : SRIPEFINPQPVVDIEMHKPKEEPGIVPALTNGTGTSSIPEPKTISENGSSMETQDKVVILEGLSTVSAHEEWTALSVSGQRPKPRYEHGATVLQDKMYV : 200
Hap_9  : SRIPEFINPQPVVDIEMHKPKEEPGIVPALTNGTGTSSIPEPKTISENGSSMETQDKVVILEGLSTVSAHEEWTALSVSGQRPKPRYEHGATVLQDKMYV : 200
Hap_10 : SRIPEFINPQPVVDIEMHKPKEEPGIVPALTNGTGTSSIPEPKTISENGSSMETQDKVVILEGLSTVSAHEEWTALSVSGQRPKPRYEHGATVLQDKMFV : 200
         SRIPEFINPQPVVDIEMHKPKEEPGIVPALTNGTGTSSIPEPKTISENGSSMETQDKVVILEGLSTVSAHeEWTALSVSGQRPKPRYEHGATVLQDKM5V

                   *       220         *       240         *       260         *       280         *       300
Hap_1  : FGGNHNGRYLSDLQVLDLKSLTWSKIDAKLQAGTSDSAKTAQVSPCAGHSLISCGNKFFSVAGHTKDPSDSITVKEFDPHTCTWSIVRTYGKPPVSRGGQ : 300
Hap_2  : FGGNHNGRYLSDLQVLDLKSLTWSKIDAKLQAGTSDSAKTAQVSPCAGHSLISCGNKFFSVAGHTKDPSDSITVKEFDPHTCTWSIVRTYGKPPVSRGGQ : 300
Hap_3  : FGGNHNGRYLSDLQVLDLKSLTWSKIDAKLQAGTSDSAKTAQVSPCAGHSLISCGNKFFSVAGHTKDPSDSITVKEFDPHTCTWSIVRTYGKPPVSRGGQ : 300
Hap_4  : FGGNHNGRYLSDLQVLDLKSLTWSKIDAKLQAGTSDSAKTAQVSPCAGHSLISCGNKFFSVAGHTKDPSDSITVKEFDPHTCTWSIVRTYGKPPVSRGGQ : 300
Hap_5  : FGGNHNGRYLSDLQVLDLKSLTWSKIDAKLQAGTSDSAKTAQVSPCAGHSLISCGNKFFSVAGHTKDPSDSITVKEFDPHTCTWSIVRTYGKPPVSRGGQ : 300
Hap_6  : FGGNHNGRYLSDLQVLDLKSLTWSKIDAKLQAGTSDSAKTAQVSPCAGHSLISCGNKFFSVAGHTKDPSDSITVKEFDPHTCTWSIVRTYGKPPVSRGGQ : 300
Hap_7  : FGGNHNGRYLSDLQVLDLKSLTWSKIDAKLQAGTSDSAKTAQVSPCAGHSLISCGNKFFSVAGHTKDPSDSITVKEFDPHTCTWSIVRTYGKPPVSRGGQ : 300
Hap_8  : FGGNHNGRYLSDLQVLDLKSLTWSKIDAKLQAGTSDSAKTAQVSPCAGHSLISCGNKFFSVAGHTKDPSDSITVKEFDPHTCTWSIVRTYGKPPVSRGGQ : 300
Hap_9  : FGGNHNGRYLSDLQVLDLKSLTWSKIDAKLQAGTSDSAKTAQVSPCAGHSLISCGNKFFSVAGHTKDPSDSITVKEFDPHTCTWSIVRTYGKPPVSRGGQ : 300
Hap_10 : FGGNHNGRYLSDLQVLDLKSLTWSKIDAKLQAGTSDSAKTAQVSPCAGHSLISCGNKFFSVAGHTKDPSDSITVKEFDPHTCTWSIVRTYGKPPVSRGGQ : 300
         FGGNHNGRYLSDLQVLDLKSLTWSKIDAKLQAGTSDSAKTAQVSPCAGHSLISCGNKFFSVAGHTKDPSDSITVKEFDPHTCTWSIVRTYGKPPVSRGGQ
```

```
                 *       320         *       340         *       360         *       380         *       400
Hap_1  : SVTLVGTTLVVFGGEDAKRCLLNDLHILDLETMTWDDVDAIGTPPAPRSDHVAACHADRYLLIFGGGSHATCFNDLHVLDLQTMEWSRPKQQGPIPSPRA : 400
Hap_2  : SVTLVGTTLVVFGGEDAKRCLLNDLHILDLETMTWDDVDAIGTPPAPRSDHVAACHADRYLLIFGGGSHATCFNDLHVLDLQTMEWSRPKQQGPIPSPRA : 400
Hap_3  : SVTLVGTTLVVFGGEDAKRCLLNDLHILDLETMTWDDVDAIGTPPAPRSDHVAACHADRYLLIFGGGSHATCFNDLHVLDLQTMEWSRPKQQGPIPSPRA : 400
Hap_4  : SVTLVGTTLVVFGGEDAKRCLLNDLHILDLETMTWDDVDAIGTPPAPRSDHVAACHADRYLLIFGGGSHATCFNDLHVLDLQTMEWSRPKQQGPIPSPRA : 400
Hap_5  : SVTLVGTTLVVFGGEDAKRCLLNDLHILDLETMTWDDVDAIGTPPAPRSDHVAACHADRYLLIFGGGSHATCFNDLHVLDLQTMEWSRPKQQGPIPSPRA : 400
Hap_6  : SVTLVGTTLVVFGGEDAKRCLLNDLHILDLETMTWDDVDAIGTPPAPRSDHVAACHADRYLLIFGGGSHATCFNDLHVLDLQTMEWSRPKQQGPIPSPRA : 400
Hap_7  : SVTLVGTTLVVFGGEDAKRCLLNDLHILDLETMTWDDVDAIGTPPAPRSDHVAACHADRYLLIFGGGSHATCFNDLHVLDLQTMEWSRPKQQGPIPSPRA : 400
Hap_8  : SVTLVGTTLVVFGGEDAKRCLLNDLHILDLETMTWDDVDAIGTPPAPRSDHVAACHADRYLLIFGGGSHATCFNDLHVLDLQTMEWSRPKQQGPIPSPRA : 400
Hap_9  : SVTLVGTTLVVFGGEDAKRCLLNDLHILDLETMTWDDVDAIGTPPAPRSDHVAACHADRYLLIFGGGSHATCFNDLHVLDLQTMEWSRPKQQGPIPSPRA : 400
Hap_10 : SVTLVGTTLVVFGGEDAKRCLLNDLHILDLETMTWDDVDAIGTPPAPRSDHVAACHADRYLLIFGGGSHATCFNDLHVLDLQTMEWSRPKQQGPIPSPRA : 400
         SVTLVGTTLVVFGGEDAKRCLLNDLHILDLETMTWDDVDAIGTPPAPRSDHVAACHADRYLLIFGGGSHATCFNDLHVLDLQTMEWSRPKQQGPIPSPRA

                 *       420         *       440         *       460         *       480         *       500
Hap_1  : GHAGATVGENWYIVGGGNNKSGVSETLALNMSTLAWSVVSTVEGRVPLASEGMTLLYSNYSGEDYLISFGGYNGRYNNEVYALKLSVNLDFQSSTQDQAT : 500
Hap_2  : GHAGATVGENWYIVGGGNNKSGVSETLALNMSTLAWSVVSTVEGRVPLASEGMTLLYSNYSGEDYLISFGGYNGRYNNEVYALKLSVNLDLQSSTQDQAT : 500
Hap_3  : GHAGATVGENWYIVGGGNNKSGVSETLALNMSTLAWSVVSTVEGRVPLASEGMTLLYSNYSGEDYLISFGGYNGRYNNEVYALKLSVNLDLQSSTQDQAT : 500
Hap_4  : GHAGATVGENWYIVGGGNNKSGVSETLALNMSTLAWSVVSTVEGRVPLASEGMTLLYSNYSGEDYLISFGGYNGRYNNEVYALKLSVNLDLQSSTQDQAT : 500
Hap_5  : GHAGATVGENWYIVGGGNNKSGVSETLALNMSTLAWSVVSTVEGRVPLASEGMTLLYSNYSGEDYLISFGGYNGRYNNEVYALKLSVNLDLQSSTQDQAT : 500
Hap_6  : GHAGATVGENWYIVGGGNNKSGVSETLALNMSTLAWSVVSTVEGRVPLASEGMTLLYSNYSGEDYLISFGGYNGRYNNEVHALKLSVNLDLQSSTQDQAT : 500
Hap_7  : GHAGATVGENWYIVGGGNNKSGVSETLALNMSTLAWSVVSTVEGRVPLASEGMTLLYSNYSGEDYLISFGGYNGRYNNEVYALKLSVNLDLQSSTQDQAT : 500
Hap_8  : GHAGATVGENWYIVGGGNNKSGVSETLALNMSTLAWSVVSTVEGRVPLASEGMTLLYSNYSGEDYLISFGGYNGRYNNEVYALKLSVNLDLQSSTQDQAT : 500
Hap_9  : GHAGATVGENWYIVGGGNNKSGVSETLALNMSTLAWSVVSTVEGRVPLASEGMTLLYSNYSGEDYLISFGGYNGRYNNEVYALKLSVNLDLQSSTQDQAT : 500
Hap_10 : GHAGATVGENWYIVGGGNNKSGVSETLALNMSTLAWSVVSTVEGRVPLASEGMTLLYSNYSGEDYLISFGGYNGRYNNEVYALKLSVNLDLQSSTQDQAT : 500
         GHAGATVGENWYIVGGGNNKSGVSETLALNMSTLAWSVVSTVEGRVPLASEGMTLLYSNYSGEDYLISFGGYNGRYNNEVyALKLSVNLDlQSSTQDQAT

                 *       520         *       540         *       560         *       580         *       600
Hap_1  : SDSTSRVLEPEVEISQDGKIREIAMDNADSKNRNDEANEQLLAALKAEKEELEATLNREGLQTVQLKEEITEAEARNAELTKELQSVRGQLAAEQSRCFK : 600
Hap_2  : SDSTSRVLEPEVEISQDGKIREIAMDNADSKNRNDEANEQLLAALKAEKEELEATLNREGLQTVQLKEEITEAEARNAELTKELQSVRGQLAAEQSRCFK : 600
Hap_3  : SDSTSRVLEPEVEISQDGKIREIAMDNADSKNRNDEANEQLLAALKAEKEELEATLNREGLQTVQLKEEITEAEARNAELTKELQSVRGQLAAEQSRCFK : 600
Hap_4  : SDSTSRVLEPEVEISQDGKIREIAMDNADSKNRNDEANEQLLAALKAEKEELEATLNREGLQTVQLKEEITEAEARNAELTKELQSVRGQLAAEQSRCFK : 600
Hap_5  : SDSTSRVLEPEVEISQDGKIREIAMDNADSKNRNDEANEQLLAALKAEKEELEATLNREGLQTVQLKEEITEAEARNAELTKELQSVRGQLAAEQSRCFK : 600
Hap_6  : SDSTSRVLEPEVEISQDGKIREIAMDNADSKNRNDEANEQLLAALKAEKEELEATLNREGLQTVQLKEEITEAEARNAELTKELQSVRGQLAAEQSRCFK : 600
Hap_7  : SDSTSRVLEPEVEISQDGKIREIAMDNADSKNRNDEANEQLLAALKAEKEELEATLNREGLQTVQLKEEITEAEARNAELTKELQSVRGQLAAEQSRCFK : 600
Hap_8  : SDSTSRVLEPEVEISQDGKIREIAMDNADSKNRNDEANEQLLAALKAEKEELEATLNREGLQTVQLKEEITEAEARNAELTKELQSVRGQLAAEQSRCFK : 600
Hap_9  : SDSTSRVLEPEVEISQDGKIREIAMDNADSKNRNDEANEQLLAALKAEKEELEATLNREGLQTVQLKEEITEAEARNAELTKELQSVRGQLAAEQSRCFK : 600
Hap_10 : SDSTSRVLEPEVEISQDGKIREIAMDNADSKNRNDEANEQLLAALKAEKEELEATLNREGLQTVQLKEEITEAEARNAELTKELQSVRGQLAAEQSRCFK : 600
         SDSTSRVLEPEVEISQDGKIREIAMDNADSKNRNDEANEQLLAALKAEKEELEATLNREGLQTVQLKEEITEAEARNAELTKELQSVRGQLAAEQSRCFK
```

```
                  *       620         *       640         *       660         *
Hap_1  : LEVDVAELRQKLQSLDALEREVELLRRQKAASEQEQAALDAKQKKQAGSGGVWGWLVGTPPDDDDSESS* : 669
Hap_2  : LEVDVAELRQKLQSLDALEREVELLRRQRAASEQEQAALDAKQKKQAGSGGVWGWLVGTPPDDDDSESS* : 669
Hap_3  : LEVDVAELRQKLQSLDALEREVELLRRQKAASEQEQAALDAKQKKQAGSGGVWGWLVGTPPDDDDSESS* : 669
Hap_4  : LEVDVAELRQKLQSLDALEREVELLRRQKAASEQEQAALDAKQKKQAGSGGVWGWLVGTPPDDDDSESS* : 669
Hap_5  : LEVDVAELRQKLQSLDALEREVELLRRQKAASEQEQAALDAKQKKQAGSGGVWGWLVGTPPDDDDSESS* : 669
Hap_6  : LEVDVAELRQKLQSLDALEREVELLRRQKAASEQEQAALDAKQKKQAGSGGVWGWLVGTPPDDDDSESS* : 669
Hap_7  : LEVDVAELRQKLQSLDALEREVELLRRQKAASEQEQAALDAKQKKQAGSGGVWGWLVGTPPDDDDSESS* : 669
Hap_8  : LEVDVAELRQKLQSLDALEREVELLRRQKAASEQEQAALDAKQKKQAGSGGVWGWLVGTPPDDDDSESS* : 669
Hap_9  : LEVDVAELRQKLQSLDALEREVELLRRQKAASEQEQAALDAKQKKQAGSGGVWGWLVGTPPDDDDSESS* : 669
Hap_10 : LEVDVAELRQKLQSLDALEREVELLRRQKAASEQEQAALDAKQKKQAGSGGVWGWLVGTPPDDDDSESS* : 669
         LEVDVAELRQKLQSLDALEREVELLRRQ4AASEQEQAALDAKQKKQAGSGGVWGWLVGTPPDDDDSESS
```

Supplementary Data 2. Protein sequences of HvACBP7 in different haplotypes

Alignment between haplotypes of barley HvACBP7 proteins. Black indicates that the amino acid residues of the ten haplotypes at this site are the same
