# Supplementary material for: Acyl-CoA-binding protein (ACBP) genes involvement in response to abiotic stress and exogenous hormone application in barley (Hordeum vulgare L.)
Source: BMC Plant Biol. 2024 Apr 2;24:236. doi: 10.1186/s12870-024-04944-6 (PMC10985865; doi:10.1186/s12870-024-04944-6)
Supplement: Supplementary file 4 — Supplementary Material 4 [file 12870_2024_4944_MOESM4_ESM.pdf]

```
                     *        20         *        40         *        60         *        80         *
Hap_1 : MGLKEEFEEYAEKAKTLPDTTTNESKLCLYSLYKQATVGPVNTDRPGLFDLAGKAKWDAWKSVEAKSKEEAMADYITKVKQLLEEAAAASASS* : 93
Hap_2 : MGLKEEFEEYAEKAKTLPDTTTNESKLCLYSLYKQATVGPVNTDRPGLFDLAGKAKWDAWKSVEAKSKEEAMADYITKVKQLLEEAAAASASS* : 93
Hap_3 : MGLKEEFEEYAEKAKTLPDTTTNESKLCLYSLYKQATVGPVNTDRPGLFDLAGKAKWDAWKSVEAKSKEEAMADYITKVKQLLEEAAAASASS* : 93
Hap_4 : MGLKEEFEEYAEKAKTLPDTTTNESKLCLYSLYKQATVGPVNTDRPGLFDLAGKAKWDAWKSVEAKSKEEAMADYITKVKQLLEEAAAASASS* : 93
Hap_5 : MGLKEEFEEYAEKAKTLPDTTTNESKLCLYSLYKQATVGPVNTDRPGLFDLAGKAKWDAWKSVEAKSKEEAMADYITKVKQLLEEAAAASASS* : 93
Hap_6 : MGLKEEFEEYAEKAKTLPDTTTNESKLCLYSLYKQATVGPVNTDRPGLFDLAGKAKWDAWKSVEAKSKEEAMADYITKVKQLLEEAAAASASS* : 93
Hap_7 : MGLKEEFEEYAEKAKTLPDTTTNESKLCLYSLYKQATVGPVNTDRPGLFDLAGKAKWDAWKSVEAKSKEEAMADYITKVKQLLEEAAAASASS* : 93
Hap_8 : MGLKEEFEEYAEKAKTLPDTTTNESKLCLYSLYKQATVGPVNTDRPGLFDLAGKAKWDAWKSVEAKSKEEAMADYITKVKQLLEEAAAASASS* : 93
Hap_9 : MGLKEEFEEYAEKAKTLPDTTTNESKLCLYSLYKQATVGPVNTDRPGLFDLAGKAKWDAWKSVEAKSKEEAMADYITKVKQLLEEAAAASASS* : 93
        MGLKEEFEEYAEKAKTLPDTTTNESKLCLYSLYKQATVGPVNTDRPGLFDLAGKAKWDAWKSVEAKSKEEAMADYITKVKQLLEEAAAASASS
```

Supplementary Data 4. Protein sequences of HvACBP8 in different haplotypes

Alignment between haplotypes of barley HvACBP8 proteins. Black means that the amino acid residues of the nine haplotypes at this site are the same
