# Supplementary material for: Acyl-CoA-binding protein (ACBP) genes involvement in response to abiotic stress and exogenous hormone application in barley (Hordeum vulgare L.)
Source: BMC Plant Biol. 2024 Apr 2;24:236. doi: 10.1186/s12870-024-04944-6 (PMC10985865; doi:10.1186/s12870-024-04944-6)
Supplement: Supplementary file 5 — Supplementary Material 5 [file 12870_2024_4944_MOESM5_ESM.pdf]

a

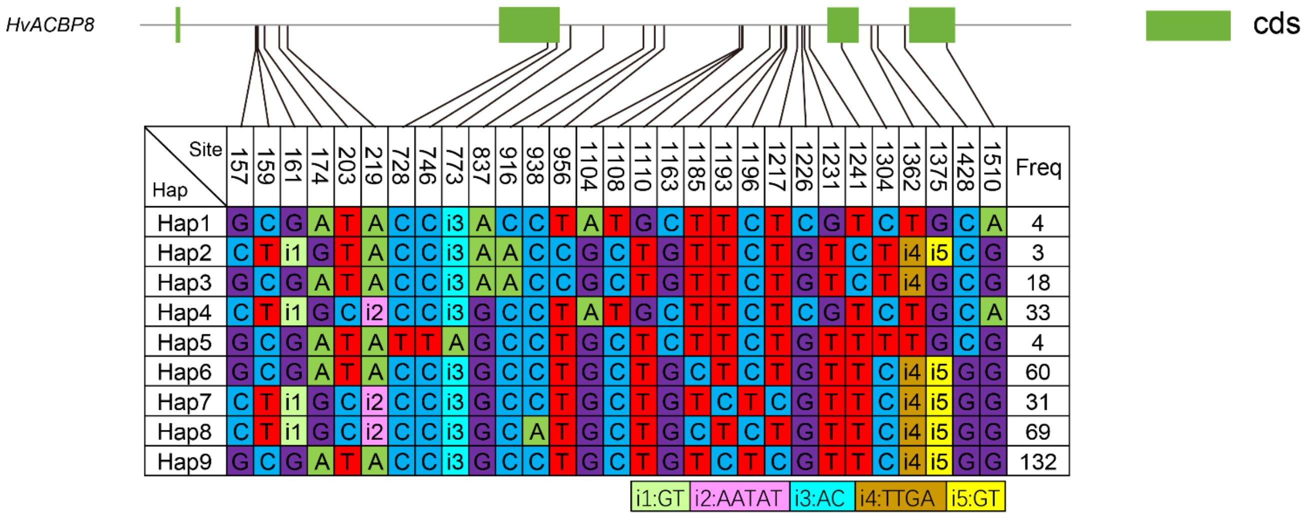

Hap \ Site	157	159	161	174	203	219	728	746	773	837	916	938	956	1104	1108	1110	1163	1185	1193	1196	1217	1226	1231	1241	1304	1362	1375	1428	1510	Freq
Hap1	G	C	G	A	T	A	C	C	i3	A	C	C	T	A	T	G	C	T	T	C	T	C	G	T	C	T	G	C	A	4
Hap2	C	T	i1	G	T	A	C	C	i3	A	A	C	C	G	C	T	G	T	T	C	T	G	T	C	T	i4	i5	C	G	3
Hap3	G	C	G	A	T	A	C	C	i3	A	A	C	C	G	C	T	G	T	T	C	T	G	T	C	T	i4	G	C	G	18
Hap4	C	T	i1	G	C	i2	C	C	i3	G	C	C	T	A	T	G	C	T	T	C	T	C	G	T	C	T	G	C	A	33
Hap5	G	C	G	A	T	A	T	T	A	G	C	C	T	G	C	T	C	T	T	C	T	G	T	T	T	T	G	C	G	4
Hap6	G	C	G	A	T	A	C	C	i3	G	C	C	T	G	C	T	G	C	T	C	T	G	T	T	C	i4	i5	G	G	60
Hap7	C	T	i1	G	C	i2	C	C	i3	G	C	C	T	G	C	T	G	T	C	T	C	G	T	T	C	i4	i5	G	G	31
Hap8	C	T	i1	G	C	i2	C	C	i3	G	C	A	T	G	C	T	G	C	T	C	T	G	T	T	C	i4	i5	G	G	69
Hap9	G	C	G	A	T	A	C	C	i3	G	C	C	T	G	C	T	G	T	C	T	C	G	T	T	C	i4	i5	G	G	132

i1:GT | i2:AATAT | i3:AC | i4:TTGA | i5:GT

b

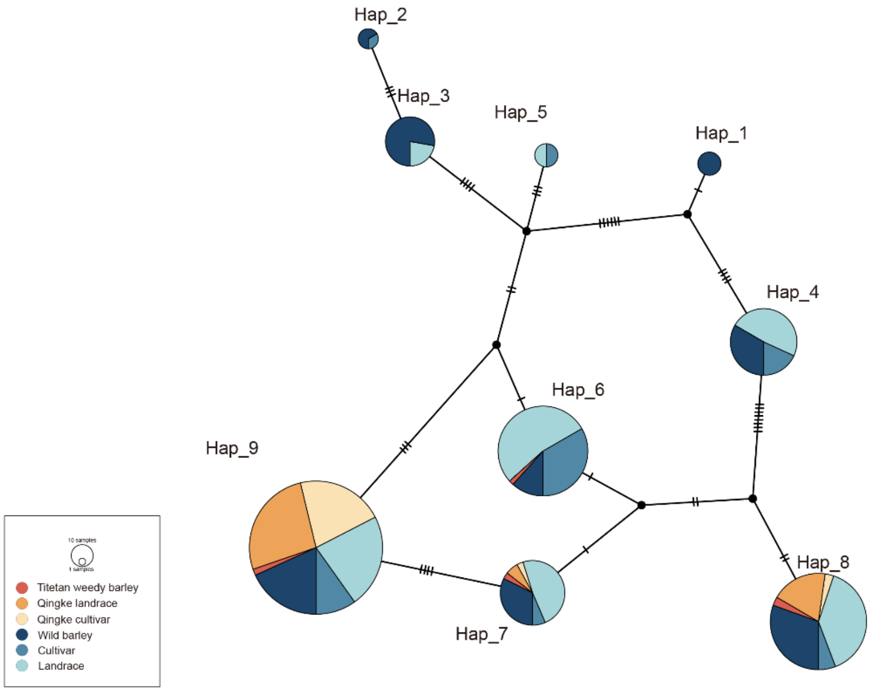

Supplementary Data 3：Haplotype analysis of *HvACBP8*

(a) Single-nucleotide polymorphisms (SNPs) were identified for haplotype analysis. Light-green rectangles: exons, Straight lines: introns, SNPs in the promoter and coding sequence are shown in the upper table. Indels are represented by i1–5. (b) Proportions of each barley resource having each haplotype, with haplotype network analysis.
